# m^6^A Modifications Play Crucial Roles in Glial Cell Development and Brain Tumorigenesis

**DOI:** 10.3389/fonc.2021.611660

**Published:** 2021-02-24

**Authors:** Jing Wang, Yongqiang Sha, Tao Sun

**Affiliations:** ^1^ Center for Precision Medicine, School of Medicine and School of Biomedical Sciences, Huaqiao University, Xiamen, China; ^2^ College of Materials Science and Engineering, Huaqiao University, Xiamen, China

**Keywords:** *N*^6^-methyladenosine (m^6^A), brain development, neural stem cell, glial cell, brain tumor, glioma

## Abstract

RNA methylation is a reversible post-transcriptional modification to RNA and has a significant impact on numerous biological processes. *N* ^6^-methyladenosine (m^6^A) is known as one of the most common types of eukaryotic mRNA methylation modifications, and exists in a wide variety of organisms, including viruses, yeast, plants, mice, and humans. Widespread and dynamic m^6^A methylation is identified in distinct developmental stages in the brain, and controls development of neural stem cells and their differentiation into neurons, glial cells such as oligodendrocytes and astrocytes. Here we summarize recent advances in our understanding of RNA methylation regulation in brain development, neurogenesis, gliogenesis, and its dysregulation in brain tumors. This review will highlight biological roles of RNA methylation in development and function of neurons and glial cells, and provide insights into brain tumor formation, and diagnostic and treatment strategies.

## Introduction

**Figure 1:**
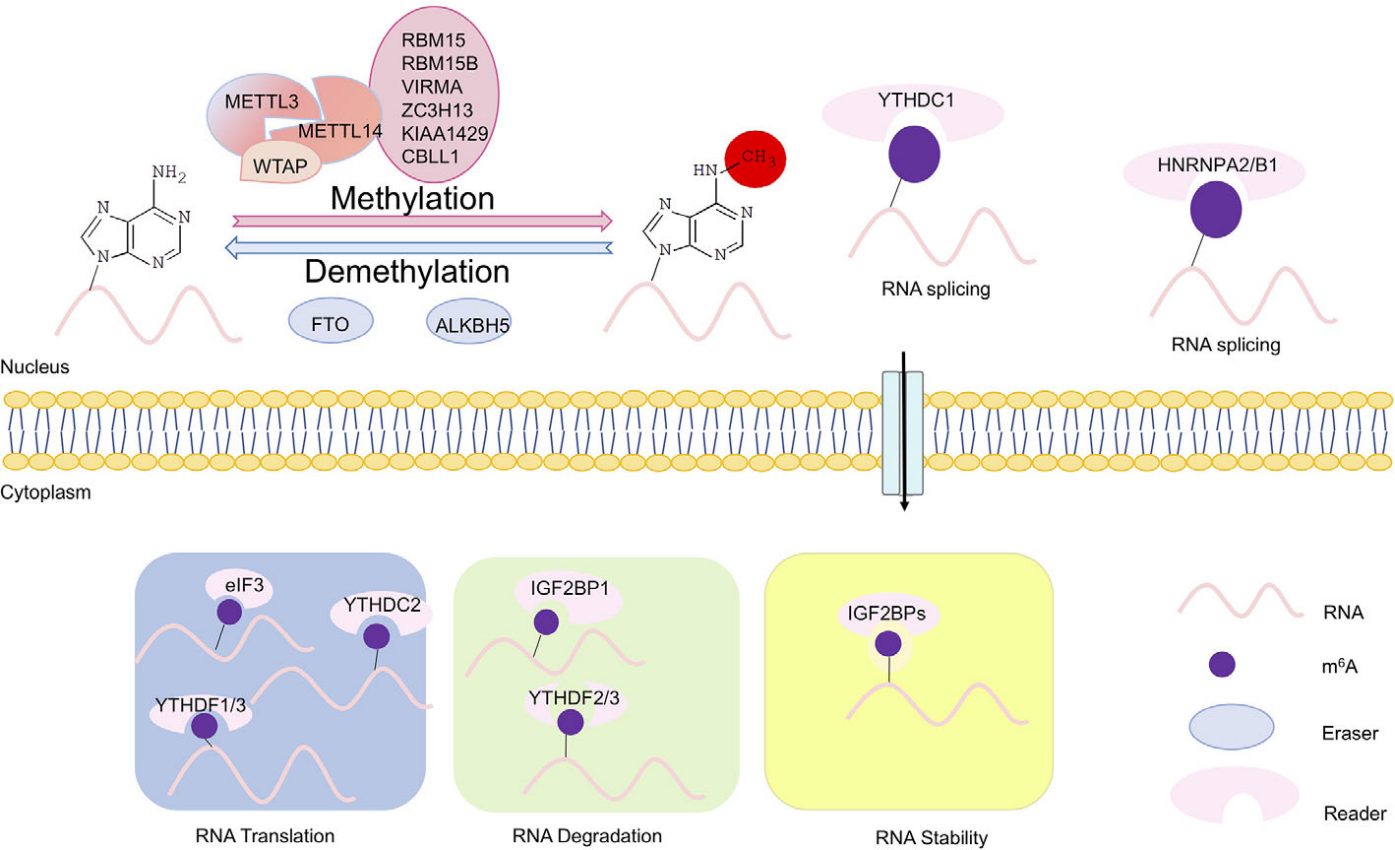
Scheme of m^6^A modifications. The writers, erasers, and readers of *N*6-methyladenosine (m^6^A). The m^6^A writer complex, which comprises the core methyltransferase-like protein 3 (METTL3) and its adaptors, is located in the nucleus. m^6^A demethylation is executed by two demethylases FTO and ALKBH5. The m^6^A erasers also are localized in the nucleus. In the nucleus, m^6^A can bind specific nuclear reader proteins such as YTHDC1 and HNRNPA2/B1, which may affect RNA splicing and mRNA export. Upon mRNA being exported to the cytoplasm, m^6^A binds to specific reader proteins, which affects stability, translation and/or localization of mRNAs. In the cytoplasm, translation of m^6^A modified mRNAs is mediated by the m^6^A readers YTHDF1, YTHDF3, and YTHDC2, the eukaryotic translation initiation factor eIF3, and METTL3. YTHDF2 and YTHDF3 regulate degradation of m^6^A modified mRNAs, while the insulin-like growth factor 2 mRNA-binding proteins (IGF2BPs) enhances stability m^6^A modified mRNAs.

*N* ^6^-methyladenosine (m^6^A) is the most common and abundant methylation modification in RNA molecules present in eukaryotes (1, 2). More than 150 distinct chemical marks on cellular RNAs have been identified to date, and m^6^A modifications account for over 80% of all RNA methylations (3). High-throughput m^6^A sequencing studies have shown that thousands of mRNAs and non-coding RNAs are modified by m^6^A, which in turn affects gene expression, participates in animal development and pathogenesis of human diseases (4, 5). m^6^A is the most prevalent internal mRNA modification, with an average of one to three modifications per transcript, and potentially regulates every step in mRNA metabolism to some extent (6).

m^6^A methylation is catalyzed by an m^6^A methyltransferase complex (MTC) composed of methyltransferase-like 3 and 14 (METTL3 and METTL14) and their cofactors such as Wilms tumor 1-associated protein (WTAP), termed as “writer” (7–9). Removal of m^6^A is facilitated by Fat mass and obesity-associated (FTO) and AlkB homolog H5 (ALKBH5), two m^6^A demethylases that recognize distinct sets of target mRNAs, termed as “eraser” (10, 11). YTHDF1/2/3 and YTHDC1, members of the YT521-B homology (YTH) domain family proteins, are m^6^A direct “readers,” which affect translation, stability, and splicing of target mRNAs (12) (**Figure 1**). m^6^A modification has emerged as a multifaceted controller for gene expression regulation, mediated through its effector proteins—writers, readers, and erasers (6).

m^6^A modifications in mRNAs or non-coding RNAs play important roles in virtually all types of bioprocesses including tissue development, self-renewal and differentiation of stem cells, heat shock response, circadian clock control, DNA damage response, and maternal-to-zygotic transition (8, 12). m^6^A is an important epitranscriptomic mark with high abundance in the central nervous system (CNS), and plays a crucial role in neural development and function (13). Dysregulation of m^6^A modifications also is associated with tumorigenesis of various cancers, such as gliomas (14).

In this review, we first summarize the recent advance in our understanding of biological functions and underlying molecule mechanisms of m^6^A regulation in neural development, with an emphasis in neurons and glial cells. We then highlight m^6^A regulatory roles in formation of brain tumors.

## *N* ^6^-methyladenosine (m^6^A) Modifications

As the most common and prevalent internal modification in eukaryotic mRNAs, m^6^A methylation has a significant impact on various physiological events (6, 15).

### Components and Functions of m^6^A Modifications

Modification of m^6^A on mRNAs is post-transcriptionally installed, erased, and recognized by m^6^A methyltransferases, demethylases and m^6^A-specific binding proteins, respectively. Methyltransferases include METTL3/14, WTAP, RBM15/15B, and KIAA1429, also termed as “writers” (1, 7, 9) (**Figure 1**). METTL3 is the catalytic subunit, and METTL14 is an essential component to facilitate RNA binding (16). m^6^A methyltransferase is widely conserved among eukaryotic species that range from yeast, plants, and flies to mammals (17, 18). Demethylases consist of FTO and ALKBH5, termed as “erasers” (10, 11, 19). And m^6^A-specific binding proteins include YTHDF1/2/3 and IGF2BP1, termed as “readers” (20) (**Figure 1**).

In mammals, m^6^A is widely distributed in multiple tissues, with a higher expression in the liver, kidney, and brain than in other tissues (21). In the rodent brain, the global level of m^6^A is developmentally regulated, with expression peaking in the adult brain (22). Studies of m^6^A modifications have revealed m^6^A binding sites in over 25% of human transcripts, with enrichment in long exons, near stop codon and 3′ untranslated terminal region (3’-UTR) (2, 21, 22).

m^6^A modification in eukaryotic mRNAs exhibits substantial contributions to post-transcriptional gene expression regulation, and plays crucial and evolutionarily conserved roles in fundamental cellular processes such as meiosis and cell differentiation in yeast, plants, and mammals (18). m^6^A methyltransferase is crucial for yeast meiosis, differentiation of mouse embryonic stem cells, and viability of human cells (18, 23, 24). Depletion of the METTL3 homologs in yeast and flies leads to developmental arrest and defects in gametogenesis (18, 25). The m^6^A demethylase AlkBH5 deficient male mice are characterized by impaired fertility, resulting from apoptosis that affects meiotic metaphase-stage spermatocytes (19). Moreover, m^6^A modifications improve the stability of mRNAs and can control protein production (15, 26). For example, YTHDF1/2/3 exhibit 5- to 20-fold higher binding affinity for methylated RNAs compared to unmethylated RNAs (12). YTHDF1 and YTHDF3 bind m^6^A at the 3′ end of transcripts and increase their cap-dependent translation, possibly through a looping interaction with eukaryotic elongation factor 3 (eIF3) (15, 27). And the mRNA-binding protein IGF2BP1 enhances stability and translation of oncogenic mRNAs, including c-Myc, and in turn promotes cell proliferation and tumorigenesis (28).

### m^6^A Modifications in mRNAs

m^6^A modification appears to directly affect biological activities of RNAs with unclear molecular mechanisms (**Figure 1**). m^6^A modification directly recruits m^6^A-specific proteins of the YTH domain family (29). These proteins contribute methyl-selective RNA binding with an amount of cellular processes, and produce m^6^A-dependent regulation of pre-mRNA processing, microRNA (miRNA) processing, translation initiation, and mRNA decay (5). Mature mRNAs with m^6^A methylation are regulated in the cytoplasm by the YTH family proteins. YTHDF1 is associated with initiating ribosomes, and delivers its target mRNAs for enhanced translation efficiency in HeLa cells (15). A second YTH family protein, YTHDF2, directly recruits the CCR4-NOT deadenylase complex and accelerates degradation of methylated transcripts (12, 30).

Moreover, some RNA transcripts exhibit increased half-lives upon m^6^A methylation. The well-established RNA stabilizer protein (HuR)/microRNA pathway mediates m^6^A-upregulated RNA stability (8). m^6^A modifications can assist protein binding either by destabilizing the helix around it, in turn allowing protein access, or by causing a conformation change to place m^6^A in a single-stranded context (31).

### m^6^A Modifications in Translational Regulations

Interestingly, the methyltransferase complex may also function as a protein scaffold in RNA-processing and metabolism (19, 32). Translation regulation by m^6^A occurs during initiation and elongation. Sequences in the 5’-UTR of mRNAs are important for ribosome recruitment and translation initiation (33). m^6^A residues within 5’-UTR can act as an m^6^A-induced ribosome engagement site (MIRES), which promotes cap-independent translation of mRNAs (34). Moreover, eukaryotic elongation factor 3 family (eIF3a/b/h) can function as m^6^A readers, and physically interact with METTL3 to enhance translation by forming densely packed polyribosomes through recognizing m^6^A modifications at the 5’-UTR of mRNAs (35, 36). METTL3, independent of METTL14, is associated with chromatin and localized to the transcriptional start sites of active genes that have the CAATT-box binding protein CEBPZ present. Promoter-bound METTL3 can induce m^6^A modifications within the coding region of the associated mRNA transcript, and enhance its translation by relieving ribosome stalling (34, 37). In addition, METTL13-mediated methylation of eukaryotic elongation factor 1A (eEF1A) increases translation elongation and enhances protein synthesis to promote tumorigenesis (38).

Based on above biochemical and genetic evidence, m^6^A methylation plays a broad role in many aspects of bioprocesses by direct modifications on mRNAs, and through regulating RNA transcription and translation.

## m^6^A Modifications in nervous system development

Proper development of the brain is critical for its function. Deficits in neural development have been implicated in many brain disorders. In the adult mouse brain, almost half of stably expressed RNAs are methylated, indicating important roles of m^6^A in brain development and function.

### m^6^A Modifications in Development of the Cerebral Cortex

**Figure 2:**
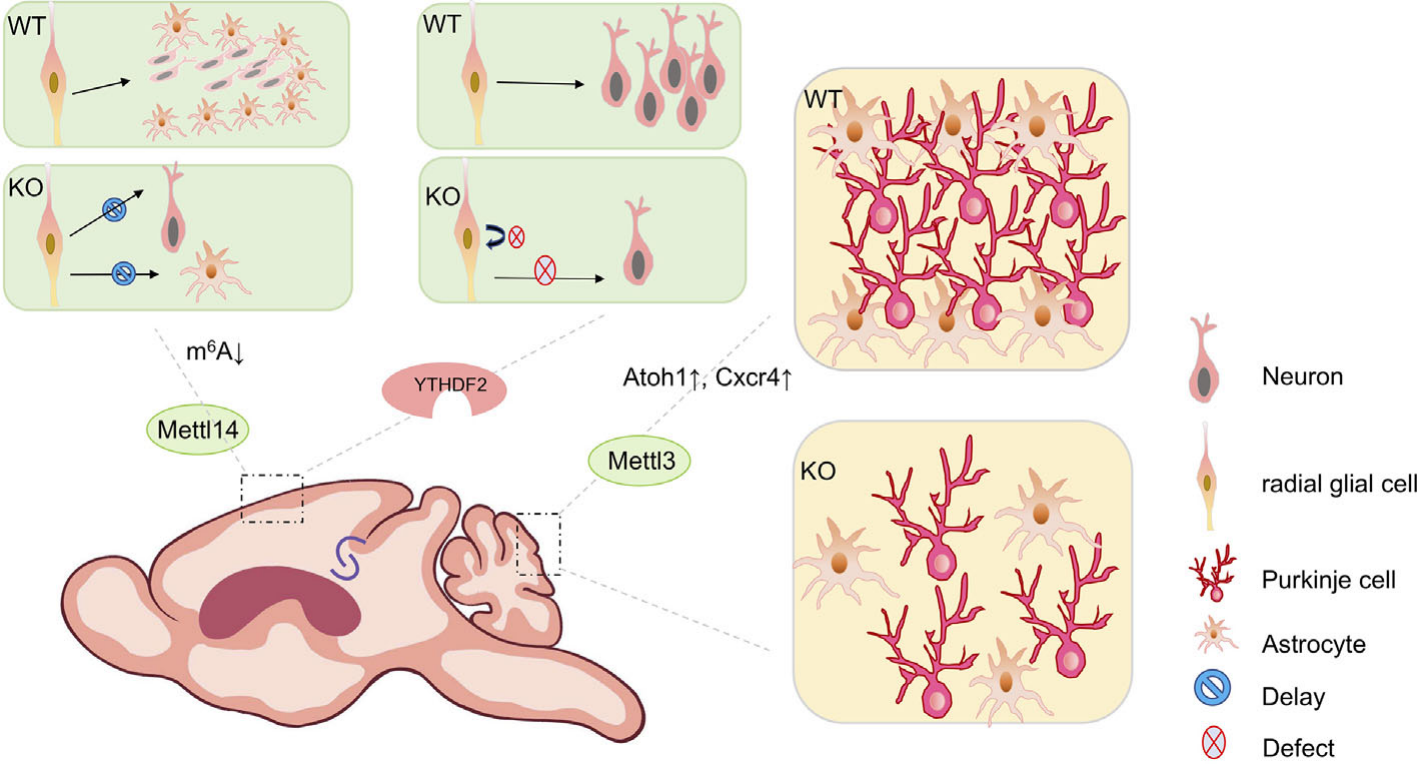
The biological impact of m^6^A modifications in mouse brain development. m^6^A modifications play a key role throughout brain development during mouse embryonic and postnatal stages. *Mettl14* conditional knockout mouse (cKO) in the mouse embryonic brain causes prolonged cell cycle in cortical radial glial cells, results in delayed neurogenesis and gliogenesis, compared to wild type (WT) mice. Conditional depletion of *Ythdf2* in mice causes decreased self-renewal of neural stem/progenitor cells (NSPCs) and defects in spatiotemporal generation of neurons in the embryonic cortex. Knocking out *Mettl3* in the mouse embryonic brain causes cerebellar hypoplasia. Ectopic expression of *Mettl3* leads to a disorganized laminal structure of both Purkinje cells and glial cells. Key developmental genes such as *Atoh1* and *Cxcr4* are abnormally upregulated due to the extended mRNA half-lives induced by m^6^A depletion.

The cerebral cortex controls social interactions, decision-making, behavioral output, and other complex cognitive behaviors (39). In the developing cortex, m^6^A modifications are enriched in transcripts involved in neurogenesis and neuronal differentiation (40, 41). Studies have shown that *Mettl14* deletion leads to a significant reduction of m^6^A levels in cortical mRNAs *in vivo* and in cultured cortical neural progenitors (40). *Mettl14* deletion in the embryonic mouse brain causes prolonged cell cycle in cortical radial glia cells (RGCs), results in delayed neurogenesis and gliogenesis (40) (**Figure 2**).

Moreover, *Fto* knockout mice show a significant increase of m^6^A levels in transcripts of hippocampus (42). Altered expression of genes with m^6^A modifications contributes to impaired adult neurogenesis (42, 43). In addition, conditional depletion of *Ythdf2* in mice causes decreased self-renewal of neural stem/progenitor cells (NSPCs) and defects in spatiotemporal generation of neurons in the embryonic cortex (44). *Ythdf1* knockout mice exhibit impaired hippocampal synaptic transmission and long-term potentiation (13). *Ythdf1* re-expression in hippocampus in adult *Ythdf1* knockout mice rescues behavioral and synaptic defects, while hippocampus-specific acute knockdown of *Ythdf1* or *Mettl3* recapitulates the hippocampal deficiency (13) (**Figure 2**).

### m^6^A Modifications in Cerebellar Development

Studies have shown that m^6^A levels are higher in the cerebellum than in the cerebral cortex, and a substantial number of cerebellar RNAs exhibits developmentally regulated methylation (45). m^6^A writers (METTL3, METTL14, and WTAP) and erasers (ALKBH5 and FTO) are highly expressed at the early stage of cerebellar development by postnatal day 7 (P7), and show a gradual reduction towards the maturation of cerebellar neurons by P60 (45). From P7 to P60, numbers of temporal-specific m^6^A peaks in start codon regions of RNA transcripts are greatly increased, while they are decreased in the coding sequence (CDS) and stop codon regions, which suggests that m^6^A modification status might be associated with cerebellar development (45).

Knocking out *Mettl3* in the mouse embryonic brain causes cerebellar hypoplasia, due to drastically enhanced apoptosis of newborn cerebellar granule cells (CGCs) in the external granular layer (EGL) (46) (**Figure 2**). Key developmental genes such as *Atoh1* and *Cxcr4* are abnormally upregulated due to the extended mRNA half-lives induced by m^6^A depletion (46). Ectopic expression of *Mettl3* leads to a disorganized laminal structure of both Purkinje cells and glial cells (45). Moreover, deletion of the eraser gene *Alkbh5* causes increased nuclear export of hypermethylated RNAs, and abnormal proliferation and differentiation in the cerebellum (45). In addition, the cerebellum of *Fto*-deficient mouse is smaller than that of wild-type mouse (42).

### m^6^A Modifications in Synaptogenesis and Axon Guidance

m^6^A modifications also contribute to neuronal growth and regeneration as well as to the local regulation of synaptic functions (22, 47). Synaptic m^6^A epitranscriptome (SME), which is functionally enriched in synthesis and modulation of tripartite synapses, has been identified in mouse adult forebrains using low-input m^6^A-sequencing of synaptosomal RNAs (48). The synaptic m^6^A peak distribution along mRNAs shows characteristic accumulation at the stop codon (22, 40).

Increased adenosine methylation in a subset of mRNAs important for neuronal signaling, including many in the dopaminergic (DA) signaling pathway has been found in the midbrain and striatum of *Fto*-knockout mice (43). Inhibition of FTO leads to increased m^6^A modifications and decreased local translation of axonal *GAP-43* mRNA, which eventually represses axon elongation (49). Moreover, knockdown of *Ythdf1* in hippocampal neurons reduces the cell surface expression of AMPA receptor subunit GluA1 and causes altered spine morphology and reduced excitatory synaptic transmission (48). Mutation of m^6^A sites in *Robo3.1* mRNA or *YTHDF1* knockdown or knockout leads to reduction of Robo3.1 protein, but not *Robo3.1* mRNA, indicating that YTHDF1-mediated translation of m^6^A-modified *Robo3.1* mRNA controls pre-crossing of axon guidance in the spinal cord (50). In addition, *YTHDF3*-knockdown neurons display a decreased percentage of spines containing a postsynaptic density (PSD) and surface GluA1 expression, indicating synaptic deficits in both structure and transmission (48).

In summary, these studies demonstrate important functions of m^6^A modifications in the nervous system. Mechanistic roles of m^6^A in regulating proliferation and differentiation of neural progenitors remain unclear. Whether such a mechanism is widespread within the brain will be an important area of future research.

## m^6^A Modifications in Glial Cell Development

Glial cells, including oligodendrocytes and astrocytes, which are derived from the neuroepithelium in the CNS, and microglia, which are derived from mesodermal hematopoietic cells, make up 10–20% of the cells in the *Drosophila* nervous system and at least 50% of the cells in the human brain (51).

### m^6^A Regulations in Gliogenesis

Embryonic neurogenesis and gliogenesis involve NSC proliferation, differentiation of NSCs into various neural and glial cell types, and their migration to their final destinations in the nervous system.

In the developing mouse cortex, NSCs or RGCs initially give rise to neurons in embryonic stages, and later switch to produce glial cells in early postnatal stages (52). Recent studies have shown that epigenetic mechanisms are involved in the precise spatiotemporal gene expression program, which controls transition in the developmental competence of progenitor cells in the sequential generation of neural and glial progeny and the maintenance of their differentiated identities (53). Several studies have investigated the mechanisms by which m^6^A regulates RGC differentiation. Reduction of m^6^A level decreases RGC proliferation, resulting in delayed neurogenesis and gliogenesis (40). *Mettl3* depletion not only inhibits neuronal proliferation and differentiation, but also interferes differentiation of NSCs towards the glial lineage (54).

### m^6^A Regulation of Oligodendrocytes

Transcripts that encode a number of histone modifiers are dynamically marked by m^6^A in oligodendrocytes precursor cells (OPCs) and oligodendrocytes, suggesting that m^6^A RNA modifications may play a role in regulating the expression of epigenetic modifiers in distinct oligodendrocyte lineages (55). Inactivating an m^6^A writer component METTL14 results in unchanged numbers of OPCs, decreased numbers of oligodendrocytes and hypomyelination in the CNS (56). A number of RNA transcripts that encode transcription factors implicated in oligodendrocytes lineage progression is dynamically marked by m^6^A at different stages of the oligodendrocyte lineage. *Mettl14* ablation disrupts postmitotic oligodendrocyte maturation and has distinct effects on transcriptomes of OPCs and oligodendrocytes (56). Moreover, loss of *Mettl14* in oligodendrocyte lineage cells causes aberrant splicing of myriad RNA transcripts, including those that encode the essential paranodal component neurofascin 155 (NF155) (56). These results indicate a time-specific post-transcriptional regulatory role of m^6^A in OPCs and oligodendrocytes.

Moreover, studies have shown that m^6^A reader PRRC2A controls OPC generation, proliferation, and fate determination. Deletion of *Prrc2a* in mouse OPCs leads to hypomyelination and consequent locomotive and cognitive defects, without affecting neurogenesis (57). PRRC2A binds and stabilizes the methylated transcript of oligodendrocyte transcription factor 2 (*Olig2*), a key oligodendroglial lineage determination transcription factor, in an m^6^A-dependent manner (57).

### m^6^A Regulation of Astrocytes

Studies have shown that astrocytes and neurons are derived from a common neuroepithelial precursor (58). *Mettl3* regulates lineage commitment during NSC differentiation, with a preference towards a neuronal fate. Mettl3-mediated m^6^A modification reduces the percentage of new born astrocytes (54). Knockout of *Mettl14* in the mouse developing nervous system results in a significant decrease in the number of S100b^+^ astrocytes (40). Knocking down *Mettl3* causes reduced astrocyte numbers in the developing cerebellum (45). *Alkbh5* deficiency leads to reduced dendritic arborization of Purkinje cells, concomitant with an increase in disorganization of the radial fibers in astrocytes (45).

In summary, these results indicate that m^6^A modifications are critical for proper temporal progression of gliogenesis including oligodendrocytes and astrocytes.

## m^6^A Modifications in Primary Brain tumors

Brain tumors are categorized into various types based on their nature, origin, rate of growth, and progression stage (59). Primary brain tumors can be broadly classified as malignant or non-malignant (benign) tumors, and graded from I to IV using a classification scheme specified by the World Health Organization (WHO) (60). Glioblastoma (GBM), a grade IV glioma, is the most prevalent (80% of all brain tumors) malignant and lethal intrinsic tumor in the CNS (61, 62).

RNA modifications, especially m^6^A modifications, have been shown to be essential for tumor development (63, 64). In particular, m^6^A modifications seem to play pivotal roles since both m^6^A writers and erasers contribute to the tumorigenesis of glioblastoma, especially glioma stem cells (GSCs) (62). Studies have shown that as the WHO grade is increased, the expression of WTAP, RBM15, YTHDF, and ALBKH5 is increased, while the expression of FTO is decreased in glioma (65).

### m^6^A Writers Play an Oncogenic Role in Glioblastoma

**Figure 3:**
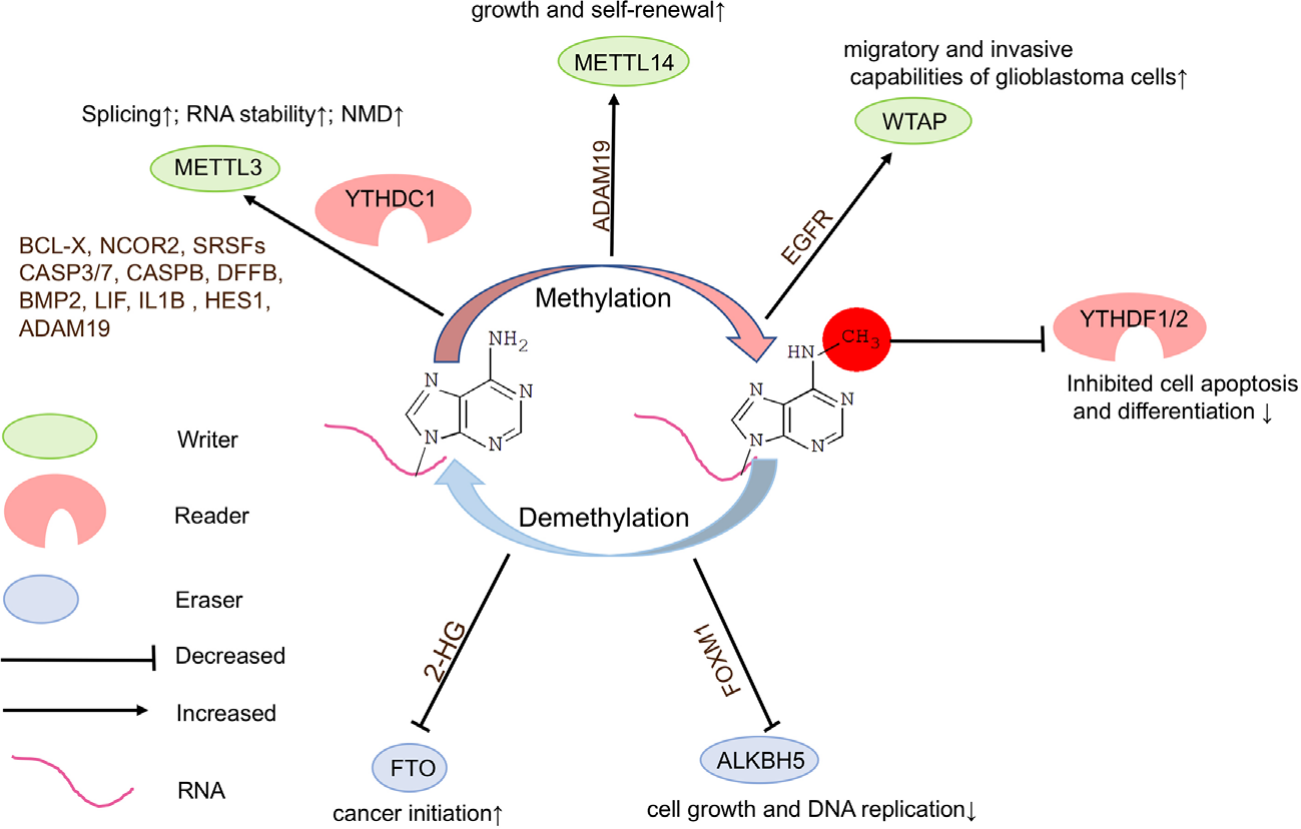
Functions of m^6^A modifications in brain tumors. *N*6-methyladenosine (m^6^A) modifications play a crucial role in brain tumorigenesis. Inadequate or dysregulated expression of writers, erasers, and readers of m^6^A is associated with brain tumor formation.

Studies have shown that high expression of *METTL3* is associated with clinical aggressiveness of malignant gliomas. METTL3 plays an oncogenic role by modulating nonsense-mediated mRNA decay (NMD) of splicing factors and alternative splicing of *BCLX* and *NCOR2* isoform switches in glioblastoma (66). Silencing *METTL3* or overexpressing dominant-negative mutant form of *METTL3* suppresses growth and self-renewal of GSCs. METTL3 maintains the stability of a specific set of transcripts, such as apoptosis pathways and glial differentiation genes including *SRSF1/2/3/6/11*, *CASP3/7*, *CASPB*, *DFFB*, *BMP2*, *LIF*, *IL1B*, and *HES1* in glioblastoma (66) (**Figure 3**). It appears that the oncogenic ability of METTL3 is dependent upon its methyltransferase catalytic domain. Knockdown of METTL14 expression reduces m^6^A levels in transcripts in GSCs, however, knockout of *METTL14* has no effect on tumorigenesis of glioblastoma, suggesting that catalytic activity in METTL3 might be crucial in tumorigenesis (21, 67).

In addition, low levels of METTL3 or METTL14 lead to decreased m^6^A modifications on *ADAM19* and increased level of *ADAM19* in GSCs, ultimately causing glioma (67). The elevated sphere-formation rate induced by knockdown of *Mettl3* or *Mettl14* in GSCs can be reversed by knockdown of *ADAM19*, suggesting that *ADAM19* acts as a target of m^6^A RNA methylation to regulate GSC self-renewal. It appears that knockdown of *Mettl3* or *Mettl14* dramatically promotes human GSC growth, self-renewal, and tumorigenesis (67). These controversial discoveries suggest that the role of METTL3 in glioblastoma requires further studies based on large amount of tumor samples and well-designed experimental systems.

Moreover, WTAP, an important component of the m^6^A methyltransferase complex, can regulate migratory and invasive capabilities of glioblastoma cells by increasing expression of epidermal growth factor receptor (*EGFR*) (68) (**Figure 3**).

### Suppressing m^6^A Erasers May Inhibit Tumorigenesis

In glioma, mutations occur only in 0.1% of cases for m^6^A ereasers ALKBH5 and no mutations have been reported in FTO (69). Knockdown of *Alkbh5* inhibits cell growth and decreased DNA replication in GSCs, and causes decreased *Foxm1* transcription, and extended survival with a lower rate of tumor formation in mice (70) (**Figure 3**). These results demonstrate that the demethylation activity of ALKBH5 is critical to represses GSC-induced tumorigenesis.

Moreover, mutation of isocitrate dehydrogenase 1 (*IDH1*) occurs frequently, which results in accumulation of the metabolic byproduct 2-hydroxy-glutarate (2-HG) in glioma. 2-HG can inhibit FTO activity, and in turn increase global m^6^A modifications and contribute to cancer initiation (66) (**Figure 3**). In addition, treatment of GSCs with an FTO inhibitor MA2 suppresses GSC-initiated tumorigenesis and prolongs the lifespan of GSC-engrafted mice (67). Studies also have shown that FTO may play an oncogenic role *via* maintaining the stability of transcripts of avian myelocytomatosis viral oncogene homolog (c-Myc) and CCAAT enhancer binding protein alpha (CEBPA) in glioma, especially IDH1/2 mutant glioma (71).

### m^6^A Readers Promote Progression of Glioblastoma

Studies have shown that *YTHDF1* and *YTHDF2* mRNA expression levels are positively correlated with malignancy of gliomas, with significant increases in higher grade gliomas, suggesting a role for these m^6^A readers in glioma progression (65, 69) (**Figure 3**). YTHDF2 may recognize specific methylated mRNAs, lead to their decay and subsequently to decreased cell apoptosis and differentiation, and in turn promote glioblastoma growth and de-differentiation, and also stabilize *MYC* and *VEGFA* transcripts in GSCs in an m^6^A-dependent manner (12, 72).

Moreover, a major splicing factor serine and arginine rich splicing factor 3 (SRSF3) is frequently upregulated in clinical glioma specimens (73). Knockdown of *YTHDC1* leads to accumulation of NMD of *SRSF3* mRNAs in glioblastoma cells, which can accelerate the proliferation of tumor cells (66).

In summary, altered m^6^A modifications are associated with the occurrence and development of glioblastoma, likely through regulating self-renewal of glioma stem cells. It appears that both m^6^A writers and erasers play an oncogenic role, and m^6^A readers function in progression in development of glioblastoma. However, inconsistent results indicate complicity of m^6^A modifications in brain tumor formation, likely through regulating distinct downstream genes.

## Conclusions and Perspectives

Brain development is based on coordinated spatiotemporal cell fate decisions, and tightly regulated gene expression. Accumulating studies have shown that m^6^A methylation plays an important role in brain development and even in brain tumorigenesis. A major challenge is to identify specific target RNAs for m^6^A modifications in specific cell types and at different developmental stages. Recent improvements to m^6^A mapping methods will undoubtedly facilitate studies of activity-dependent changes to the epitranscriptome within distinct RNA populations in the brain. Moreover, an interesting research will be to determine whether changes to the mRNA modification landscape are causing factors or a consequence of activity-dependent regulation of gene expression.

How m^6^A methylation regulates brain tumor formation remains obscure. Taking the advantage of technical development of m^6^A methylation analysis at the single-cell level, mechanistic understanding of RNA methylation in different cell types will be revealed. Interestingly, in the late stage of glioma, high m^6^A modification levels may increase epigenetic reprogramming of non-GSCs into GSCs, whereas knockdown of *METTL3* may reduce the ratio of GSCs in glioblastoma (66). Thus, a more profound breakthrough in the role of m^6^A methylation in brain tumor diagnostics and treatment strategy also should be developed.

## Author Contributions

JW and TS wrote the manuscript and produced figures. YQS modified the manuscript. TS edited the manuscript. All authors contributed to the article and approved the submitted version.

## Funding

This work was supported by the Scientific Research Funds of Huaqiao University (Z16Y0017, TS), and the National Natural Science Foundation of China (31771141 and 81701132).

## Conflict of Interest

The authors declare that the research was conducted in the absence of any commercial or financial relationships that could be construed as a potential conflict of interest.

## References

[1] Schwartz S Mumbach MR Jovanovic M Wang T Maciag K Bushkin GG. Perturbation of m6A writers reveals two distinct classes of mRNA methylation at internal and 5’ sites. Cell Rep (2014) 8(1):284–96. 10.1016/j.celrep.2014.05.048 PMC4142486 24981863

[2] Li J Yang X Qi Z Sang Y Liu Y Xu B. The role of mRNA m(6)A methylation in the nervous system. Cell Biosci (2019) 9(1). 10.1186/s13578-019-0330-y PMC6701067 31452869

[3] Boccaletto P Machnicka MA Purta E Piatkowski P Baginski B Wirecki TK. MODOMICS: a database of RNA modification pathways. 2017 update. Nucleic Acids Res (2018) 46(D1):D303–7. 10.1093/nar/gkx1030 PMC5753262 29106616

[4] Hsu PJ Shi H He C. Epitranscriptomic influences on development and disease. Genome Biol (2017) 18(1):197. 10.1186/s13059-017-1336-6 29061143 PMC5654102

[5] Roundtree IA Evans ME Pan T He C. Dynamic RNA Modifications in Gene Expression Regulation. Cell (2017) 169(7):1187–200. 10.1016/j.cell.2017.05.045 PMC5657247 28622506

[6] Shi H Wei J He C. Where, When, and How: Context-Dependent Functions of RNA Methylation Writers, Readers, and Erasers. Mol Cell (2019) 74(4):640–50. 10.1016/j.molcel.2019.04.025 PMC6527355 31100245

[7] Liu J Yue Y Han D Wang X Fu Y Zhang L. A METTL3-METTL14 complex mediates mammalian nuclear RNA N6-adenosine methylation. Nat Chem Biol (2014) 10(2):93–5. 10.1038/nchembio.1432 PMC3911877 24316715

[8] Wang Y Li Y Toth JI Petroski MD Zhang Z Zhao JC. N6-methyladenosine modification destabilizes developmental regulators in embryonic stem cells. Nat Cell Biol (2014) 16(2):191–8. 10.1038/ncb2902 PMC4640932 24394384

[9] Ping XL Sun BF Wang L Xiao W Yang X Wang WJ. Mammalian WTAP is a regulatory subunit of the RNA N6-methyladenosine methyltransferase. Cell Res (2014) 24(2):177–89. 10.1038/cr.2014.3 PMC3915904 24407421

[10] Zhao BS Roundtree IA He C. Post-transcriptional gene regulation by mRNA modifications. Nat Rev Mol Cell Biol (2017) 18(1):31–42. 10.1038/nrm.2016.132 27808276 PMC5167638

[11] Jia G Fu Y Zhao X Dai Q Zheng G Yang Y. N6-methyladenosine in nuclear RNA is a major substrate of the obesity-associated FTO. Nat Chem Biol (2011) 7(12):885–7. 10.1038/nchembio.687 PMC3218240 22002720

[12] Wang X Lu Z Gomez A Hon GC Yue Y Han D. N6-methyladenosine-dependent regulation of messenger RNA stability. Nature (2014) 505(7481):117–20. 10.1038/nature12730 PMC3877715 24284625

[13] Shi H Zhang X Weng YL Lu Z Liu Y Lu Z. m(6)A facilitates hippocampus-dependent learning and memory through YTHDF1. Nature (2018) 563(7730):249–53. 10.1038/s41586-018-0666-1 PMC6226095 30401835

[14] Deng X Su R Weng H Huang H Li Z Chen J. RNA N(6)-methyladenosine modification in cancers: current status and perspectives. Cell Res (2018) 28(5):507–17. 10.1038/s41422-018-0034-6 PMC5951805 29686311

[15] Wang X Zhao BS Roundtree IA Lu Z Han D Ma H. N(6)-methyladenosine Modulates Messenger RNA Translation Efficiency. Cell (2015) 161(6):1388–99. 10.1016/j.cell.2015.05.014 PMC4825696 26046440

[16] Wang X Feng J Xue Y Guan Z Zhang D Liu Z. Corrigendum: Structural basis of N(6)-adenosine methylation by the METTL3-METTL14 complex. Nature (2017) 542(7640):260. 10.1038/nature21073 28099411

[17] Horowitz S Horowitz A Nilsen TW Munns TW Rottman FM. Mapping of N6-methyladenosine residues in bovine prolactin mRNA. Proc Natl Acad Sci U S A (1984) 81(18):5667–71. 10.1073/pnas.81.18.5667 PMC391771 6592581

[18] Yue Y Liu J He C. RNA N6-methyladenosine methylation in post-transcriptional gene expression regulation. Genes Dev (2015) 29(13):1343–55. 10.1101/gad.262766.115 PMC4511210 26159994

[19] Zheng G Dahl JA Niu Y Fedorcsak P Huang CM Li CJ. ALKBH5 is a mammalian RNA demethylase that impacts RNA metabolism and mouse fertility. Mol Cell (2013) 49(1):18–29. 10.1016/j.molcel.2012.10.015 23177736 PMC3646334

[20] Muller S Glass M Singh AK Haase J Bley N Fuchs T. IGF2BP1 promotes SRF-dependent transcription in cancer in a m6A- and miRNA-dependent manner. Nucleic Acids Res (2019) 47(1):375–90. 10.1093/nar/gky1012 PMC6326824 30371874

[21] Meyer KD Patil DP Zhou J Zinoviev A Skabkin MA Elemento O. 5’ UTR m(6)A Promotes Cap-Independent Translation. Cell (2015) 163(4):999–1010. 10.1016/j.cell.2015.10.012 26593424 PMC4695625

[22] Meyer KD Saletore Y Zumbo P Elemento O Mason CE Jaffrey SR. Comprehensive analysis of mRNA methylation reveals enrichment in 3’ UTRs and near stop codons. Cell (2012) 149(7):1635–46. 10.1016/j.cell.2012.05.003 PMC3383396 22608085

[23] Schwartz S Agarwala SD Mumbach MR Jovanovic M Mertins P Shishkin A. High-resolution mapping reveals a conserved, widespread, dynamic mRNA methylation program in yeast meiosis. Cell (2013) 155(6):1409–21. 10.1016/j.cell.2013.10.047 PMC3956118 24269006

[24] Geula S Moshitch-Moshkovitz S Dominissini D Mansour AA Kol N Salmon-Divon M. Stem cells. m6A mRNA methylation facilitates resolution of naïve pluripotency toward differentiation. Science (New York NY) (2015) 347(6225):1002–6. 10.1126/science.1261417 25569111

[25] Hongay CF Orr-Weaver TL. Drosophila Inducer of MEiosis 4 (IME4) is required for Notch signaling during oogenesis. Proc Natl Acad Sci U S A (2011) 108(36):14855–60. 10.1073/pnas.1111577108 PMC3169142 21873203

[26] Wang X He C. Dynamic RNA modifications in posttranscriptional regulation. Mol Cell (2014) 56(1):5–12. 10.1016/j.molcel.2014.09.001 25280100 PMC7129666

[27] Shi H Wang X Lu Z Zhao BS Ma H Hsu PJ. YTHDF3 facilitates translation and decay of N(6)-methyladenosine-modified RNA. Cell Res (2017) 27(3):315–28. 10.1038/cr.2017.15 PMC5339834 28106072

[28] Huang H Weng H Sun W Qin X Shi H Wu H. Recognition of RNA N(6)-methyladenosine by IGF2BP proteins enhances mRNA stability and translation. Nat Cell Biol (2018) 20(3):285–95. 10.1038/s41556-018-0045-z PMC5826585 29476152

[29] Dominissini D Moshitch-Moshkovitz S Schwartz S Salmon-Divon M Ungar L Osenberg S. Topology of the human and mouse m6A RNA methylomes revealed by m6A-seq. Nature (2012) 485(7397):201–6. 10.1038/nature11112 22575960

[30] Du H Zhao Y He J Zhang Y Xi H Liu M. YTHDF2 destabilizes m(6)A-containing RNA through direct recruitment of the CCR4-NOT deadenylase complex. Nat Commun (2016) 7:12626. 10.1038/ncomms12626 27558897 PMC5007331

[31] Roost C Lynch SR Batista PJ Qu K Chang HY Kool ET. Structure and thermodynamics of N6-methyladenosine in RNA: a spring-loaded base modification. J Am Chem Soc (2015) 137(5):2107–15. 10.1021/ja513080v PMC4405242 25611135

[32] Xiao W Adhikari S Dahal U Chen YS Hao YJ Sun BF. Nuclear m(6)A Reader YTHDC1 Regulates mRNA Splicing. Mol Cell (2016) 61(4):507–19. 10.1016/j.molcel.2016.01.012 26876937

[33] Xue S Tian S Fujii K Kladwang W Das R Barna M. RNA regulons in Hox 5’ UTRs confer ribosome specificity to gene regulation. Nature (2015) 517(7532):33–8. 10.1038/nature14010 PMC4353651 25409156

[34] Zhang C Fu J Zhou Y. A Review in Research Progress Concerning m6A Methylation and Immunoregulation. Front Immunol (2019) 10:922. 10.3389/fimmu.2019.00922 31080453 PMC6497756

[35] Choe J Lin S Zhang W Liu Q Wang L Ramirez-Moya J. mRNA circularization by METTL3-eIF3h enhances translation and promotes oncogenesis. Nature (2018) 561(7724):556–60. 10.1038/s41586-018-0538-8 PMC6234840 30232453

[36] Lin S Choe J Du P Triboulet R Gregory RI. The m(6)A Methyltransferase METTL3 Promotes Translation in Human Cancer Cells. Mol Cell (2016) 62(3):335–45. 10.1016/j.molcel.2016.03.021 PMC4860043 27117702

[37] Barbieri I Tzelepis K Pandolfini L Shi J Millán-Zambrano G Robson SC. Promoter-bound METTL3 maintains myeloid leukaemia by m(6)A-dependent translation control. Nature (2017) 552(7683):126–31. 10.1038/nature24678 PMC6217924 29186125

[38] Liu S Hausmann S Carlson SM Fuentes ME Francis JW Pillai R. METTL13 Methylation of eEF1A Increases Translational Output to Promote Tumorigenesis. Cell (2019) 176(3):491–504.e421. 10.1016/j.cell.2018.11.038 30612740 PMC6499081

[39] Boles NC Temple S. Epimetronomics: m6A Marks the Tempo of Corticogenesis. Neuron (2017) 96(4):718–20. 10.1016/j.neuron.2017.11.002 29144970

[40] Yoon K-J Ringeling FR Vissers C Jacob F Pokrass M Jimenez-Cyrus D. Temporal Control of Mammalian Cortical Neurogenesis by m6A Methylation. Cell (2017) 171(4):877–89.e817. 10.1016/j.cell.2017.09.003 28965759 PMC5679435

[41] Du K Zhang L Lee T Sun T. m(6)A RNA Methylation Controls Neural Development and Is Involved in Human Diseases. Mol Neurobiol (2019) 56(3):1596–606. 10.1007/s12035-018-1138-1 29909453

[42] Li L Zang L Zhang F Chen J Shen H Shu L. Fat mass and obesity-associated (FTO) protein regulates adult neurogenesis. Hum Mol Genet (2017) 26(13):2398–411. 10.1093/hmg/ddx128 PMC6192412 28398475

[43] Hess ME Hess S Meyer KD Verhagen LA Koch L Bronneke HS. The fat mass and obesity associated gene (Fto) regulates activity of the dopaminergic midbrain circuitry. Nat Neurosci (2013) 16(8):1042–8. 10.1038/nn.3449 23817550

[44] Li M Zhao X Wang W Shi H Pan Q Lu Z. Ythdf2-mediated m(6)A mRNA clearance modulates neural development in mice. Genome Biol (2018) 19(1):69. 10.1186/s13059-018-1436-y 29855337 PMC5984442

[45] Ma C Chang M Lv H Zhang ZW Zhang W He X. RNA m(6)A methylation participates in regulation of postnatal development of the mouse cerebellum. Genome Biol (2018) 19(1):68. 10.1186/s13059-018-1435-z 29855379 PMC5984455

[46] Wang CX Cui GS Liu X Xu K Wang M Zhang XX. METTL3-mediated m6A modification is required for cerebellar development. PloS Biol (2018) 16(6):e2004880. 10.1371/journal.pbio.2004880 29879109 PMC6021109

[47] Flamand MN Meyer KD. The epitranscriptome and synaptic plasticity. Curr Opin Neurobiol (2019) 59:41–8. 10.1016/j.conb.2019.04.007 PMC6858947 31108373

[48] Merkurjev D Hong WT Iida K Oomoto I Goldie BJ Yamaguti H. Synaptic N(6)-methyladenosine (m(6)A) epitranscriptome reveals functional partitioning of localized transcripts. Nat Neurosci (2018) 21(7):1004–14. 10.1038/s41593-018-0173-6 29950670

[49] Yu J Chen M Huang H Zhu J Song H Zhu J. Dynamic m6A modification regulates local translation of mRNA in axons. Nucleic Acids Res (2018) 46(3):1412–23. 10.1093/nar/gkx1182 PMC5815124 29186567

[50] Zhuang M Li X Zhu J Zhang J Niu F Liang F. The m6A reader YTHDF1 regulates axon guidance through translational control of Robo3.1 expression. Nucleic Acids Res (2019) 47(9):4765–77. 10.1093/nar/gkz157 PMC6511866 30843071

[51] Rowitch DH Kriegstein AR. Developmental genetics of vertebrate glial-cell specification. Nature (2010) 468(7321):214–22. 10.1038/nature09611 21068830

[52] Yao B Christian KM He C Jin P Ming GL Song H. Epigenetic mechanisms in neurogenesis. Nat Rev Neurosci (2016) 17(9):537–49. 10.1038/nrn.2016.70 PMC5610421 27334043

[53] Chang M Lv H Zhang W Ma C He X Zhao S. Region-specific RNA m(6)A methylation represents a new layer of control in the gene regulatory network in the mouse brain. Open Biol (2017) 7(9):170166. 10.1098/rsob.170166 28931651 PMC5627058

[54] Chen J Zhang YC Huang C Shen H Sun B Cheng X. m(6)A Regulates Neurogenesis and Neuronal Development by Modulating Histone Methyltransferase Ezh2. Genomics Proteomics Bioinformatics (2019) 17(2):154–68. 10.1016/j.gpb.2018.12.007 PMC6620265 31154015

[55] Zhou H Wang B Sun H Xu X Wang Y. Epigenetic Regulations in Neural Stem Cells and Neurological Diseases. Stem Cells Int (2018) 2018:6087143. 10.1155/2018/6087143 29743892 PMC5878882

[56] Xu H Dzhashiashvili Y Shah A Kunjamma RB Weng YL Elbaz B. m(6)A mRNA Methylation Is Essential for Oligodendrocyte Maturation and CNS Myelination. Neuron (2020) 105(2):293–309 e295. 10.1016/j.neuron.2019.12.013 31901304 PMC7137581

[57] Wu R Li A Sun B Sun JG Zhang J Zhang T. A novel m(6)A reader Prrc2a controls oligodendroglial specification and myelination. Cell Res (2019) 29(1):23–41. 10.1038/s41422-018-0113-8 30514900 PMC6318280

[58] Rasband MN. Glial Contributions to Neural Function and Disease. Mol Cell Proteomics (2016) 15(2):355–61. 10.1074/mcp.R115.053744 PMC4739659 26342039

[59] Johnson DR Guerin JB Giannini C Morris JM Eckel LJ Kaufmann TJ. 2016 Updates to the WHO Brain Tumor Classification System: What the Radiologist Needs to Know. Radiographics (2017) 37(7):2164–80. 10.1148/rg.2017170037 29028423

[60] Ostrom QT Gittleman H Truitt G Boscia A Kruchko C Barnholtz-Sloan JS. CBTRUS Statistical Report: Primary Brain and Other Central Nervous System Tumors Diagnosed in the United States in 2011-2015. Neuro-oncology (2018) 20(suppl_4):iv1–iv86. 10.1093/neuonc/noy131 30445539 PMC6129949

[61] Ostrom QT Cioffi G Gittleman H Patil N Waite K Kruchko C. CBTRUS Statistical Report: Primary Brain and Other Central Nervous System Tumors Diagnosed in the United States in 2012-2016. Neuro-oncology (2019) 21(Suppl 5):v1–v100. 10.1093/neuonc/noz150 31675094 PMC6823730

[62] Dong Z Cui H. The Emerging Roles of RNA Modifications in Glioblastoma. Cancers (Basel) (2020) 12(3):736. 10.3390/cancers12030736 PMC7140112 32244981

[63] Huang H Weng H Chen J. m(6)A Modification in Coding and Non-coding RNAs: Roles and Therapeutic Implications in Cancer. Cancer Cell (2020) 37(3):270–88. 10.1016/j.ccell.2020.02.004 PMC7141420 32183948

[64] Thapar R Bacolla A Oyeniran C Brickner JR Chinnam NB Mosammaparast N. RNA Modifications: Reversal Mechanisms and Cancer. Biochemistry (2019) 58(5):312–29. 10.1021/acs.biochem.8b00949 30346748

[65] Chai RC Wu F Wang QX Zhang S Zhang KN Liu YQ. m(6)A RNA methylation regulators contribute to malignant progression and have clinical prognostic impact in gliomas. Aging (2019) 11(4):1204–25. 10.18632/aging.101829 PMC6402513 30810537

[66] Li F Yi Y Miao Y Long W Long T Chen S. N(6)-Methyladenosine Modulates Nonsense-Mediated mRNA Decay in Human Glioblastoma. Cancer Res (2019) 79(22):5785–98. 10.1158/0008-5472.can-18-2868 PMC7360104 31530567

[67] Cui Q Shi H Ye P Li L Qu Q Sun G. m(6)A RNA Methylation Regulates the Self-Renewal and Tumorigenesis of Glioblastoma Stem Cells. Cell Rep (2017) 18(11):2622–34. 10.1016/j.celrep.2017.02.059 PMC5479356 28297667

[68] Jin DI Lee SW Han ME Kim HJ Seo SA Hur GY. Expression and roles of Wilms’ tumor 1-associating protein in glioblastoma. Cancer Sci (2012) 103(12):2102–9. 10.1111/cas.12022 PMC7659328 22957919

[69] Ceccarelli M Barthel Floris P Malta Tathiane M Sabedot Thais S Salama Sofie R Murray Bradley A. Molecular Profiling Reveals Biologically Discrete Subsets and Pathways of Progression in Diffuse Glioma. Cell (2016) 164(3):550–63. 10.1016/j.cell.2015.12.028 PMC4754110 26824661

[70] Zhang S Zhao BS Zhou A Lin K Zheng S Lu Z. m(6)A Demethylase ALKBH5 Maintains Tumorigenicity of Glioblastoma Stem-like Cells by Sustaining FOXM1 Expression and Cell Proliferation Program. Cancer Cell (2017) 31(4):591–606.e596. 10.1016/j.ccell.2017.02.013 28344040 PMC5427719

[71] Su R Dong L Li C Nachtergaele S Wunderlich M Qing Y. R-2HG Exhibits Anti-tumor Activity by Targeting FTO/m6A/MYC/CEBPA Signaling. Cell (2018) 172(1-2):90–105.e123. 10.1016/j.cell.2017.11.031 29249359 PMC5766423

[72] Dixit D Prager BC Gimple RC Poh HX Wang Y Wu Q. The RNA m6A reader YTHDF2 maintains oncogene expression and is a targetable dependency in glioblastoma stem cells. Cancer Discov (2020). 10.1158/2159-8290.cd-20-0331 PMC8110214 33023892

[73] Song X Wan X Huang T Zeng C Sastry N Wu B. SRSF3-Regulated RNA Alternative Splicing Promotes Glioblastoma Tumorigenicity by Affecting Multiple Cellular Processes. Cancer Res (2019) 79(20):5288–301. 10.1158/0008-5472.can-19-1504 PMC6801100 31462429

